# Corticomotor Excitability Changes Associated With Freezing of Gait in People With Parkinson Disease

**DOI:** 10.3389/fnhum.2020.00190

**Published:** 2020-05-21

**Authors:** Ya-Yun Lee, Min-Hao Li, Chun-Hwei Tai, Jer-Junn Luh

**Affiliations:** ^1^School and Graduate Institute of Physical Therapy, College of Medicine, National Taiwan University, Taipei, Taiwan; ^2^Department of Physical Medicine and Rehabilitation, National Taiwan University Hospital, Taipei, Taiwan; ^3^Department of Neurology, National Taiwan University Hospital, Taipei, Taiwan; ^4^College of Education, Fu-Jen Catholic University, Taipei, Taiwan

**Keywords:** Parkinson’s disease, freezing of gait, transcranial magnetic stimulation, corticomotor excitability, gait

## Abstract

**Background and Purpose:**

Freezing of gait (FOG) is a debilitating gait disorder in people with Parkinson’s disease (PD). While various neuroimaging techniques have been used to investigate the pathophysiology of FOG, changes in corticomotor excitability associated with FOG have yet to be determined. Research to date has not concluded if changes in corticomotor excitability are associated with gait disturbances in this patient population. This study aimed to use transcranial magnetic stimulation (TMS) to investigate corticomotor excitability changes associated with FOG. Furthermore, the relationship between corticomotor excitability and gait performances would be determined.

**Methods:**

Eighteen participants with PD and FOG (PD + FOG), 15 without FOG (PD − FOG), and 15 non-disabled adults (Control) were recruited for this study. Single and paired-pulse TMS paradigms were used to assess corticospinal and intracortical excitability, respectively. Gait performance was measured by the 10-Meter-Walk test. Correlation analysis was performed to evaluate relationships between TMS outcomes and gait parameters.

**Results:**

Compared with the Control group, the PD + FOG group showed a significantly lower resting motor threshold and reduced short intracortical inhibition (SICI). Correlation analysis revealed a relationship between resting motor evoked potential and step length, and between SICI and walking velocity in the Control group. While the silent period correlated with step length in the PD − FOG group, no significant relationship was observed in the PD + FOG group.

**Discussion and Conclusion:**

Compared to the Control group, the PD + FOG group exhibited reduced corticomotor inhibition. Distinct correlations observed among the three groups suggest that the function of the corticomotor system plays an important role in mediating walking ability in non-disabled adults and people with PD − FOG, while people with PD + FOG may rely on neural networks other than the corticomotor system to control gait.

## Introduction

Freezing of gait (FOG) is a debilitating phenomenon of Parkinson disease (PD) characterized by transient incapability or difficulty to move the feet forward despite an intention to do so (Nutt et al., 2011). It has been reported that ∼54.8% of people with PD experienced freezing episodes that increased in prevalence as disease progressed (Amboni et al., 2015). Even with “ON” medication status, 38.2% of individuals with PD experienced FOG (Perez-Lloret et al., 2014). Although freezing episodes may last only a few seconds, they significantly increase the risk of falls, affect daily activities, and reduce quality of life. A comprehensive understanding of the neurophysiological changes associated with FOG may facilitate the development of more effective treatment approaches (Nutt et al., 2011).

Functional magnetic resonance imaging (fMRI) and functional near infrared spectroscopy (fNIRS) have been used to provide insights into brain activation changes associated with FOG (Vercruysse et al., 2014). It was observed that people with FOG demonstrated a decreased activation of the motor, basal ganglia and ventral attention networks when performing freezing-provoking tasks in virtual reality (Shine et al., 2013). A study using fNIRS, that more directly evaluated the changes in oxygenated hemoglobin (HbO_2_) during walking, found that HbO_2_ of the prefrontal areas increased before and during freezing episodes (Maidan et al., 2015). These studies provided valuable information regarding changes in FOG associated cortical activations; it remains unclear whether increased (or decreased) activations of brain regions represents enhanced (or reduced) neural excitability. Increased brain activation may suggest an increased inhibitory process within that region (Sack and Linden, 2003). Hence, transcranial magnetic stimulation (TMS) becomes an invaluable tool for researchers to understand the changes in corticomotor excitability associated with certain disorders or disease phenotype.

Various TMS paradigms, such as single-pulse and paired-pulse paradigms, are available to evaluate the excitability of the corticospinal tract and intracortical neuronal network within the primary motor cortex (M1) (Vucic and Kiernan, 2017). Changes in corticomotor excitability of the upper-extremity muscles have been widely studied with TMS in people with PD. The studies found that PD during “ON” medication status displayed increased excitability of the corticospinal pathway and decreased intracortical inhibition compared with non-disabled adults (Cantello et al., 1991; Valls-Sole et al., 1994; Ni and Chen, 2015). Very few studies have determined the corticomotor excitability changes of lower-extremity muscles in people with PD. Since upper-extremity and lower-extremity muscles have different descending projections and functional requirement (Tremblay and Tremblay, 2002), understanding the neurophysiological changes of lower-extremity muscles is important. Enhanced corticospinal excitability in the quadriceps muscle has been reported in persons with PD (Tremblay and Tremblay, 2002). When targeting the tibialis anterior (TA) muscle, excitability of the corticospinal tract for individuals with PD did not differ from control subjects. The only observed difference was that persons with PD had significantly lower intracortical facilitation during “OFF” medication (Vacherot et al., 2010a, b). Additionally, after pooling the data from PD (“ON” and “OFF” medication) and control subjects, it was found that changes in intracortical facilitation correlated significantly with gait velocity and stride length (Vacherot et al., 2010b). These studies provided preliminary evidence of the corticomotor abnormality in the lower-extremity muscles of PD.

Since people with FOG have greater gait disturbances than those without FOG, the corticomotor excitability may change along with the symptoms. To date, no studies have evaluated differences in corticomotor excitability of lower extremity muscles between freezers and non-freezers using TMS. Understanding the potential direction of changes in corticomotor excitability may facilitate the development of appropriate interventions. This study also aimed to investigate whether changes in corticomotor excitability would correlate with walking ability in people with FOG, since gait disturbances are one of the major causes of poor quality of life for these individuals.

## Materials and Methods

### Participants

Forty-eight subjects, including 18 participants with PD and FOG (PD + FOG), 15 individuals without FOG (PD − FOG), and 15 age-matched non-disabled control subjects (Control) participated in this study. The baseline characteristics of the participants are presented in Table 1. Before joining, the participants signed an informed consent form approved by the Institutional Review Board of National Taiwan University Hospital and completed a TMS safety questionnaire. The study procedure conformed to the World Medical Association Declaration of Helsinki. The participants were excluded if they had any contraindications or concerns for receiving TMS. The participants with PD were classified as freezers and non-freezers based on the New Freezing of Gait Questionnaire (NFOG-Q) (Nieuwboer et al., 2009). A medical history chart review verified the classification of the participants with PD.

**TABLE 1:** Clinical characteristics of the participants.

	**PD + FOG**	**PD − FOG**	**Control**	***p***
Age (years)	68.2 ± 1.8	65.1 ± 1.4	68.0 ± 1.4	0.295
Gender (male/female)	10/8	8/7	6/9	0.641
Disease duration (years)	8.2 ± 1.4	3.9 ± 0.7	–	0.009*
UPDRS-III (scores)	21.1 ± 1.7	15.1 ± 1.8	–	0.022*
Levodopa equivalent dosage (mg/day)	822.3 ± 119.4	489.7 ± 76.4	–	0.032*

Values are presented as mean ± standard error for continuous variables and frequency for categorical variables. UPDRS-III: part-III of Unified Parkinson’s Disease Rating Scale. *p < 0.05.

### TMS Assessments

All TMS assessments were performed with a double-cone coil (110 mm) connected to a paired-pulse magnetic stimulator (The Magstim^®^ BiStim^2^; The Magstim Company Ltd., Whitland, United Kingdom). The target muscle was the TA of the more affected side for participants with PD and the non-dominant side for the Control participants. After skin preparation, surface electromyographic (EMG) electrodes were placed over the TA muscle belly, and a ground electrode was placed over the lateral epicondyle of the femur. EMG signal sampling rate was 4000 Hz with 0–1000 Hz band-pass filter. TMS pulses were applied over the cortical representation area (i.e., hotspot) of the TA on the M1 to activate the corticospinal tract and generate motor evoked potentials (MEPs) (Ni and Chen, 2015). Consistent with the international guidelines (Rossini et al., 1994), the hotspot of the TA and resting motor threshold (RMT) were first determined. With single-pulse TMS, 10–15 trials of MEPs were elicited using an intensity at 130% of RMT under resting and active conditions. In the resting condition, participants were instructed to sit and relax their TA muscles with ankle dorsiflexion at 90°; under the active condition, participants were asked to conduct a low-level isometric ankle dorsiflexion contraction to touch a lever bar placed above the first metatarsal shaft. The height of the bar was adjusted to 30° of dorsiflexion for each participant (Fisher et al., 2016). Peak-to-peak MEP amplitude of each trial was recorded, and mean MEP amplitude was calculated for each condition. Cortical silent period (CSP), a period when EMG activity is suppressed for a few hundred milliseconds after MEP, was also recorded during the active muscle contraction condition (Kobayashi and Pascual-Leone, 2003).

Using the paired-pulse stimulation paradigm, short intracortical inhibition (SICI) and intracortical facilitation (ICF) were obtained. Two stimuli, the conditioning stimulus and the testing stimulus, were applied to the TA hotspot. The conditioning stimulus was set at 80% of RMT, while the testing stimulus was set at 130% of RMT (Vacherot et al., 2010b). The SICI can be obtained when the inter-stimuli intervals are tuned at 2–5 ms, while ICF is measured when the inter-stimuli intervals are set between 7 and 15 ms (Vucic and Kiernan, 2017). In this study, stimuli were applied when inter-stimulus intervals were set at 2 and 3 ms for SICI (SICI_2ms_, SICI_3ms_, respectively), and 12 and 15 ms for ICF (ICF_12ms_, ICF_15ms_, respectively). The stimulation order was randomly assigned to each subject. Peak-to-peak MEP values obtained from the paired-pulse paradigm were normalized to the single-pulse resting MEP value to determine the inhibition or facilitation of the intracortical neurons.

### Gait Performances

The 10-Meter Walk Test (10MWT) was used to evaluate walking ability. Participants were instructed to walk at a comfortable walking speed along a 10-m walkway three times. The investigator recorded the time and the number of steps that the participants took during the 10-m walk, and further calculated mean walking velocity, stride length, and cadence. Good to excellent test-retest reliability for these gait parameters have been established for individuals with PD (Lang et al., 2016).

### Statistical Analysis

Baseline characteristics of the three groups (PD + FOG, PD − FOG, and Control) were analyzed with a chi-square test and one-way analysis of variance (ANOVA) for dichotomous and continuous variables, respectively. The dataset distribution was checked before analyzing the data. Since most of the outcome measures were not normally distributed, the Kruskal-Wallis test was used to determine group differences. Dunn’s *post-hoc* tests with Bonferroni corrections were performed when a significant main effect was found. In addition to *p*-values, the effect sizes (η^2^) were calculated to determine the amount of group differences (Tomczak and Tomczak, 2014). Spearman’s rho test was used to determine the relationship between TMS outcomes and gait performance. During the correlation analysis, TMS data of SICI_2ms_ and SICI_3ms_ were pooled to represent an averaged intracortical inhibition, and ICF_12ms_ and ICF_15ms_ were averaged to represent intracortical facilitation. Only gait velocity and step length were chosen for the correlation analysis because these gait parameters were mostly affected in people with FOG. All data acquired were analyzed with IBM SPSS 22.0 software (IBM Corporation, Armonk, NY, United States) using α = 0.05 for significance.

## Results

Analysis of the clinical characteristics of the participants revealed no significant differences in age and gender among the groups. The PD + FOG group had significantly longer disease duration, greater disease severity, and higher levodopa medication usage than the PD − FOG group (Table 1).

### TMS Outcomes

The TMS outcomes are presented in Table 2. Statistical analysis revealed a significant difference in RMT among the three groups [χ^2^(2) = 6.977, *p* = 0.031, η^2^ = 0.111]. Dunn’s *post-hoc* with Bonferroni corrections revealed that the PD + FOG group had lower RMT than the control group (*p* = 0.025), while there were no differences between the PD + FOG and PD − FOG groups (*p* = 0.854) or the PD − FOG and control groups (*p* = 0.400). The three groups did not differ in resting MEP [χ^2^(2) = 0.019, *p* = 0.991, η^2^ = 0.044], active MEP [χ^2^(2) = 0.862, *p* = 0.650, η^2^ = 0.026], and CSP [χ^2^(2) = 0.701, *p* = 0.701, η^2^ = 0.029].

**TABLE 2:** Corticomotor excitability and gait performances of the three groups.

	**PD + FOG**	**PD − FOG**	**Control**	***p***	**η^2^**
**Single-pulse TMS**					
RMT (%MSO)	41.9 ± 2.4*	45.3 ± 2.4	53.4 ± 3.4	0.031	0.111
Resting MEP (μV)	1013.5 ± 206.8	1074.9 ± 245.4	1000.7 ± 177.8	0.991	0.044
Active MEP (μV)	2271.2 ± 269.0	2502.6 ± 298.3	2777.4 ± 356.5	0.566	0.026
CSP (s)	0.150 ± 0.008	0.141 ± 0.104	0.153 ± 0.014	0.701	0.029
**Paired-pulse TMS**					
SICI_2ms_ (%)	45.6 ± 6.7*	41.1 ± 9.5	19.3 ± 3.7	0.011	0.155
SICI_3ms_ (%)	47.5 ± 9.6	44.0 ± 9.5	27.9 ± 5.8	0.333	0.004
ICF_12ms_ (%)	91.4 ± 18.0	69.0 ± 13.7	109.3 ± 21.5	0.274	0.004
ICF_15ms_ (%)	94.7 ± 24.1	71.2 ± 17.2	109.1 ± 29.3	0.433	0.003
**Gait performances**					
Walking velocity (m/s)	0.91 ± 0.06*	1.09 ± 0.05	1.22 ± 0.04	0.003	0.214
Step length (m)	0.48 ± 0.03*^†^	0.60 ± 0.03	0.66 ± 0.02	<0.001	0.307
Cadence (step/s)	1.88 ± 0.04	1.81 ± 0.03	1.87 ± 0.06	0.175	0.033

Values are presented as mean ± standard error. RMT, resting motor threshold; MSO, maximal stimulator output; MEP, motor evoked potentials; CSP, cortical silent period; SICI, short-interval intracortical inhibition; ICF, intracortical facilitation. *Significant difference between PD + FOG group and Control group (p < 0.05). ^†^Significant difference between PD + FOG group and PD − FOG group (p < 0.05).

**FIGURE 1:**
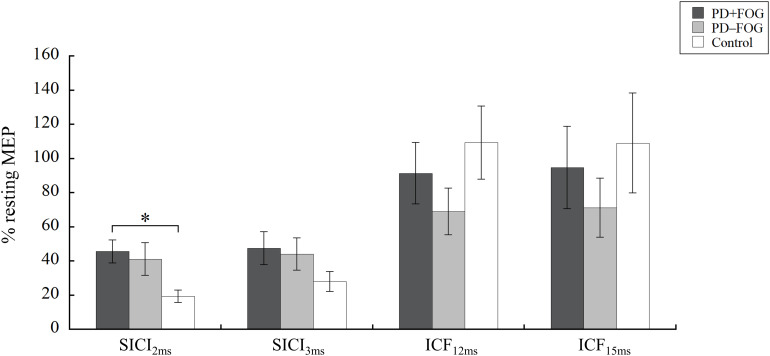
Paired-pulse TMS. Means and standard errors of the means of the PD + FOG (dark gray), PD − FOG (light gray), and Control (white) group. MEP, motor evoked potential; SICI, short intracortical inhibition; ICF, intracortical facilitation. ^∗^*P* < 0.05.

The results of the paired-pulse paradigm are shown in Figure 1. Significant group difference was found in SICI_2ms_ [χ^2^(2) = 8.993, *p* = 0.011, η^2^ = 0.155], but not in SICI_3ms_ [χ^2^(2) = 2.197, *p* = 0.333, η^2^ = 0.004], ICF_12ms_ [χ^2^(2) = 1.830, *p* = 0.400, η^2^ = 0.004], or ICF_15ms_ [χ^2^(2) = 1.869, *p* = 0.393, η^2^ = 0.003]. *Post-hoc* analysis of SICI_2ms_ showed that the PD + FOG group had larger normalized MEPs than the Control group (*p* = 0.008), but no significance was found between the PD + FOG and PD − FOG groups (*p* = 0.749) nor the PD − FOG and Control groups (*p* = 0.235). This suggests that the participants with PD + FOG had less intra-cortical inhibition at SICI_2ms_ than the Control participants.

Since baseline demographic comparison revealed that the participants with FOG group had greater disease severity with longer disease duration and higher levodopa usage than those without FOG, it was unclear whether the TMS results might be affected by these factors. To scrutinize the findings of the TMS outcomes, we re-analyzed the data by removing the individuals who had disease duration longer than 10 years and/or UPDRS III score greater than 25, leaving 13 participants in the PD − FOG group and 10 in the PD + FOG group. After removing these cases, there were no significant differences between the PD − FOG and PD + FOG groups in disease duration (*p* = 0.283), severity (*p* = 0.066), and medication usage (*p* = 0.434). When re-analyzing the TMS data of the remaining subjects, the results still showed significant group differences in RMT [χ^2^(2) = 8.602, *p* = 0.014] and SICI_2ms_ [χ^2^(2) = 7.739, *p* = 0.021], suggesting that the observed changes in corticomotor excitability were associated with FOG but not due to other factors.

### Gait Performances

Significant group differences were found for walking velocity [χ^2^(2) = 11.613, *p* = 0.003, η^2^ = 0.214]. *Post-hoc* analysis showed that participants with PD + FOG had slower walking velocity than Control subjects (*p* = 0.002), but no differences were found between the PD + FOG and PD − FOG groups (*p* = 0.215) or between the PD − FOG and Control groups (*p* = 0.381). While no group difference was found in cadence [χ^2^(2) = 3.489, *p* = 0.175, η^2^ = 0.033], a significant group difference was observed in step length [χ^2^(2) = 15.807, *p* < 0.001, η^2^ = 0.307]. *Post-hoc* analysis of step length revealed that the PD + FOG group had significantly smaller step length than the Control (*p* < 0.001) and the PD − FOG (*p* = 0.036) groups. However, the PD − FOG and Control groups did not differ significantly in step length (*p* = 0.562).

To justify the above findings, gait parameters were re-analyzed by removing the subjects who had longer disease duration, greater disease severity and higher levodopa usage as described in the previous section. Secondary analysis still revealed a significant group difference in walking velocity [χ^2^(2) = 11.959, *p* = 0.003] and step length [χ^2^(2) = 11.960, *p* = 0.003], supporting our original findings.

### Correlation Analysis

Spearman’s rho tests were performed separately for all groups (Table 3). In the Control group, significant correlation was found between walking velocity and SICI [ρ_(__13__)_ = 0.556, *p* = 0.031], while no other TMS parameters correlated with walking velocity. Step length negatively correlated with resting MEP [ρ_(__13__)_ = −0.585, *p* = 0.022]. No other correlations were observed between gait outcomes and TMS measures.

**TABLE 3:** Correlation results between gait performances and TMS outcomes.

**Correlation (ρ)**	**RMT**	**Rest MEP**	**Active MEP**	**CSP**	**SICI**	**ICF**
**Control**						
Velocity	0.240	–0.043	–0.211	0.318	0.556*	0.300
Step length	0.250	−0.585*	–0.185	–0.318	0.404	0.465
**PD − FOG**						
Velocity	0.011	–0.036	–0.061	0.399	0.504	0.013
Step length	0.126	–0.116	–0.133	0.559*	0.418	0.115
**PD + FOG**						
Velocity	–0.109	0.156	0.044	–0.133	–0.260	–0.020
Step length	–0.200	0.213	0.017	–0.126	–0.342	–0.055

Values are presented as Spearman’s rho. RMT, resting motor threshold; MEP, motor evoked potentials; CSP, cortical silent period; SICI, short-interval intracortical inhibition; ICF, intracortical facilitation. *p < 0.05.

**FIGURE 2:**
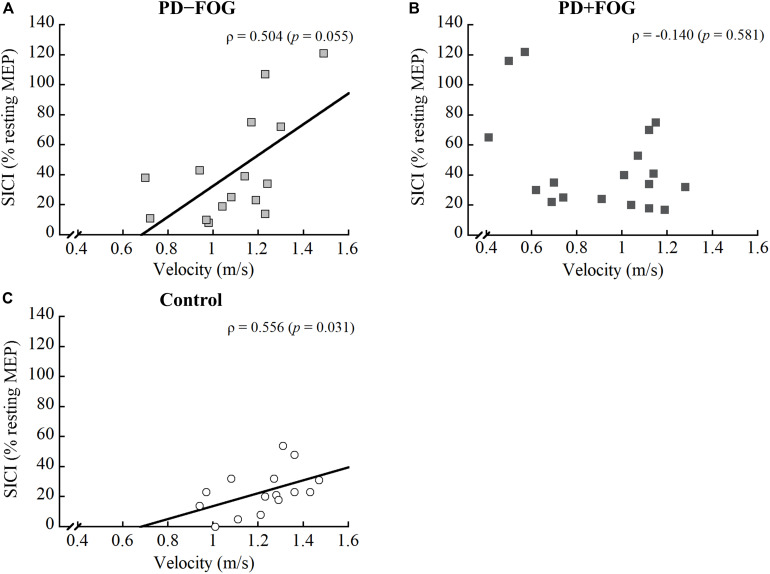
Correlation between SICI and gait velocity of the **(A)** PD − FOG, **(B)** PD + FOG, and **(C)** Control groups. SICI, short intracortical inhibition.

For the PD − FOG group, a positive correlation was observed between step length and CSP [ρ_(__13__)_ = 0.559, *p* = 0.030], while a borderline correlation was found between gait velocity and SICI [ρ_(__13__)_ = 0.504, *p* = 0.055]. No other correlations were observed between the gait parameters and TMS outcomes in this group. Different from the other two groups, there was no relationship found between the gait parameters and any of the TMS outcomes in the PD + FOG group. Since some relationships were found between SICI and velocity in the Control and PD − FOG groups but not in the PD + FOG group, Figure 2 is drawn to illustrate the different correlation patterns among the three groups.

## Discussion

This study aimed to determine the changes in corticomotor excitability in participants with PD + FOG compared to those without FOG and control subjects. Additionally, we wanted to understand whether corticomotor excitability correlates with gait performance. The results showed that, during “ON” medication, participants with PD + FOG had significantly lower RMT and reduced SICI_2ms_ than the Control subjects. Relationships was found between SICI and walking velocity, and between resting MEP and step length in the Control group. Only the CSP correlated with step length in the PD − FOG group, while no correlation was found between gait parameters and TMS outcomes in the PD + FOG group.

Resting motor threshold is an important single-pulse TMS parameter reflecting how easily the corticomotoneurons in the M1 are excited when the target muscle is at rest, and thus can represent the neuronal membrane excitability of the corticospinal tract (Vucic and Kiernan, 2017). Earlier studies conducted on upper-extremity muscles showed that RMT was similar in persons with PD and non-disabled adults, regardless of medication status (Leon-Sarmiento et al., 2013; Ni and Chen, 2015). While Vacherot et al. (2010b) did not find significant differences in RMT of the TA between the PD and Control groups (Vacherot et al., 2010b), Tremblay and Tremblay (2002) showed that individuals with PD had a significantly lower RMT of the quadriceps muscle (Tremblay and Tremblay, 2002). The inconsistent findings in RMT among the studies may be hampered by the lack of patient classification or consideration of their principal features (Cantello et al., 2002). Cantello et al. (1991) recruited participants with PD who had asymmetric body rigidity and found that RMT was lower on the rigid side than the contralateral side or than on healthy adults (Cantello et al., 1991). It was suggested that the motoneurons from the corticospinal system of the rigid side were hyperexcited, and the subliminal excitation of the alpha-motoneurons led to a lower RMT (Cantello et al., 1991, 2002). Our study provided additional evidence showing that participants with PD + FOG had significantly lower RMT than control subjects, also suggesting enhanced excitability of the corticomotor system. We could not exclude the possibility that the reduction of RMT was due to greater rigidity in people with PD + FOG even though no obvious background EMG activities were observed in these participants throughout the study. Nevertheless, appropriate patient classification may be an important factor to consider in future studies to unravel neurophysiological changes in patient populations.

Paired-pulse paradigm may be one of the most sensitive tools to highlight corticomotor abnormalities in PD (Vacherot et al., 2010a). The current study found that the PD + FOG group had significantly larger normalized MEP at SICI_2ms_ in comparison to the Control group, suggesting a decreased intracortical inhibition in people with FOG. However, no significant differences were observed between PD − FOG and Control groups. Our results partly support Vacherot et al.’s (2010b) finding of no significant differences in SICI_2ms_ of the TA muscle between people with PD and control participants regardless of medication status (Vacherot et al., 2010b). Targeting the first dorsal interosseous muscle, a significantly reduced SICI_2ms_ was found during “OFF” medication, but the reduction in intracortical inhibition was restored during the “ON” state (Ridding et al., 1995). Our study is the first to show that individuals with FOG have a reduced SICI_2ms_ of the TA muscle even during the “ON” medication status, suggesting that the intracortical inhibition is impaired in persons with PD + FOG, and dopaminergic medication could not fully restore the intracortical inhibitory control of these individuals. This reduced intracortical inhibition corresponds to the finding of decreased RMT, suggesting a compensatory hyperexcitation of the corticomotor system in people with PD + FOG. Using fMRI combined with a motor imagery of walking task, Snijders et al. (2011) found that patients with FOG, compared to those without FOG, showed a compensatory increased activation of the mesencephalic locomotor network in order to maintain motor imagery of gait (Snijders et al., 2011). Our TMS findings further supported the idea that the corticomotor system of people with FOG may be hyperexcited in order to compensate for the impaired basal ganglia.

Beyond the findings of RMT and SICI_2ms_, no statistically significant group differences were observed in other TMS variables. This is consistent with the results reported by Vacherot et al. (2010a), who also did not find any statistically significant differences in resting MEP, active MEP, CSP, and SICI_3ms_ between people with PD, regardless of “ON” or “OFF” medication, and control subjects. However, the study found impaired intracortical facilitation in the PD group during “OFF” medication, but the group difference was not evident during “ON” state. Thus, the authors suggested that dopamine substitution therapy could partially restore the function of the intracortical facilitation (Vacherot et al., 2010a, b). Since the subjects in our study were tested during “ON” medication state; it is not known whether any of the TMS variables may become impaired during the “OFF” status.

There is still a lack of comprehensive knowledge regarding how corticomotor excitability correlates with gait performance, particularly in people with PD + FOG (Swanson and Fling, 2018). Hence, the relationships between TMS outcome measures and gait variables were determined in this study. Distinct correlation patterns were observed within the three groups. For the Control group, a negative correlation was found between step length and resting MEP, while gait velocity positively correlated with the combined SICI score. As for the PD − FOG, a positive correlation was observed between step length and CSP, and a borderline correlation between walking velocity and SICI (ρ = 0.504, *p* = 0.055). The results from the PD − FOG group support those observed in the Control group. Since smaller resting MEP indicated less excited corticospinal tract and longer CSP represented greater inhibitory control of the corticospinal tract (Vucic and Kiernan, 2017), the results suggest that the modulation of appropriate step length in non-disabled adults and non-freezers relies on the inhibitory control of the corticospinal pathway. On the other hand, gait velocity correlated with SICI, indicating that walking velocity may be modulated by the intracortical inhibitory control. Individuals who had less intracortical inhibition also walked faster.

Contrary to the findings from the Control and PD − FOG groups, no significant relationships existed in the PD + FOG group. Although a good explanation for these distinct correlation patterns is lacking, we speculate that it could be related to the function or integrity of the motor circuit, connections between the sensorimotor cortex and the basal ganglia. The motor circuit plays an important role in modifying and executing motor plans during locomotion (Kesar et al., 2018). The motor circuit is presumably intact in non-disabled adults, while it is mildly to moderately affected in persons with PD − FOG. Thus, greater integrity of the motor circuit should be associated with better walking ability. However, the motor circuit is severely disrupted in people with FOG, and those individuals would rely more on the cognitive process (i.e., the frontostriatal circuit) when they intend to walk (Redgrave et al., 2010). Moreover, people with PD + FOG may use all available neural resources when executing movements. Thus, no correlation was observed in this patient population.

One limitation of this study is that participants were tested only during “ON” medication. The reason for testing the participants during the “ON” state was that persons with PD should take medication regularly. However, even with the usage of medication, many individuals, especially those with FOG, have discernable gait disturbances. Therefore, we wanted to understand whether there were any abnormalities in corticomotor excitability in persons with PD + FOG during the “ON” state. Since several earlier studies found greater changes in corticomotor excitability during “OFF” state, future studies should test the participants during “OFF” medication to understand the neurophysiological changes associated with FOG. Another limitation of the study is that the participants in the PD + FOG and PD − FOG groups were not matched in disease duration and severity. The participants were recruited using convenient sampling; thus, baseline demographic data were not fully controlled. As presented in the results, we reanalyzed the data by removing participants who had longer disease duration and greater disease severity to further scrutinize our findings. Secondary analysis still revealed significant group differences for the RMT and SICI_2ms_, supporting our initial findings. Future studies should consider disease duration- and severity-matched individuals when recruiting participants with FOG. The third limitation of the study was the potential insufficient sample size of the participants. This study recruited 33 participants with PD (18 with FOG and 15 without) according to the sample sizes being used in previous studies (Cantello et al., 2002; Tremblay and Tremblay, 2002; Vacherot et al., 2010b). A *post-hoc* power analysis revealed that the power of the study was 0.73 using SICI_2ms_ or 0.89 using velocity as the primary outcome. Nevertheless, more sample size in the future studies will facilitate the generalization of this study. Lastly, we did not pool the data of all participants with PD when performing the correlation analysis because we were more interested in understanding the association of corticomotor excitability and gait performance in each subgroup population. Investigating the relationships within each subgroup may provide additional insights regarding how the central nervous system modulates walking ability in healthy and pathological brains.

## Conclusion

Using TMS to explore the changes in corticomotor excitability associated with FOG in people with PD, it was found that freezers had a lower RMT and a reduced SICI_2ms_ when compared with non-disabled adults. Additionally, gait performances appeared to correlate with some TMS outcomes in non-disabled adults and people with PD − FOG, but not in people with PD + FOG. These findings may add a piece of information for unraveling the pathophysiology of FOG.

## Data Availability Statement

The datasets generated for this study are available on request to the corresponding author.

## Ethics Statement

The studies involving human participants were reviewed and approved by National Taiwan University Hospital Research Ethics Committee. The patients/participants provided their written informed consent to participate in this study.

## Author Contributions

Y-YL conceived and designed the study, performed the experiment, conducted the statistical analysis, and drafted the manuscript. M-HL conducted the statistical analysis and helped to draft the manuscript. C-HT helped coordinate the study and reviewed the manuscript. J-JL helped conceive the study and reviewed the manuscript.

## Conflict of Interest

The authors declare that the research was conducted in the absence of any commercial or financial relationships that could be construed as a potential conflict of interest.
